# Queueing arrival and release mechanism for K^+^ permeation through a potassium channel

**DOI:** 10.1007/s12576-019-00706-4

**Published:** 2019-08-27

**Authors:** Takashi Sumikama, Shigetoshi Oiki

**Affiliations:** 1 Department of Molecular Physiology and Biophysics, Faculty of Medical Sciences, University of Fukui, Fukui, 910-1193 Japan; 2 Present Address: Nano Life Science Institute (WPI-NanoLSI), Kanazawa University, Kakuma-machi, Kanazawa, 920-1192 Japan; 3 Present Address: Biomedical Imaging Research Center, University of Fukui, Fukui, 910-1193 Japan

**Keywords:** Molecular dynamics, Single-file permeation, Knock-on, Event-oriented analysis, Queueing mechanism

## Abstract

The mechanism underlying ion permeation through potassium channels still remains controversial. K^+^ ions permeate across a narrow selectivity filter (SF) in a single file. Conventional scenarios assume that K^+^ ions are tightly bound in the SF, and, thus, they are displaced from their energy well by ion–ion repulsion with an incoming ion. This tight coupling between entering and exiting ions has been called the “knock-on” mechanism. However, this paradigm is contradicted by experimental data measuring the water–ion flux coupling ratio, demonstrating fewer ion occupancies. Here, the results of molecular dynamics simulations of permeation through the KcsA potassium channel revealed an alternative mechanism. In the aligned ions in the SF (an ion queue), the outermost K^+^ was readily and spontaneously released toward the extracellular space, and the affinity of the relevant ion was ~ 50 mM. Based on this low-affinity regime, a simple queueing mechanism described by loose coupling of entering and exiting ions is proposed.

**Electronic supplementary material:**

The online version of this article (10.1007/s12576-019-00706-4) contains supplementary material, which is available to authorized users.

## Introduction

**Fig. 1:**
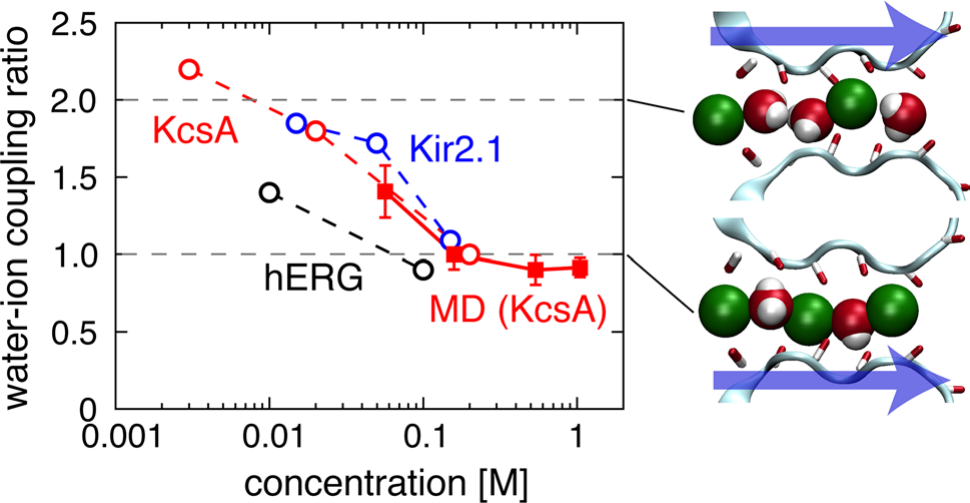
Water–ion coupling ratio (*CR*_w–i_) as a function of the K^+^ concentration. Experimental *CR*_w–i_ values as a function of [K^+^], taken from the literature, for three different potassium channels are shown: the human ether-à-go–go-Related Gene (hERG) [27], the inward rectifier (Kir2.1) [29], and the KcsA [28] channels (open circles). The concentration indicates the [K^+^] on both sides of the membrane. Our simulated *CR*_w–i_ values are superimposed (red filled squares). *CR*_w–i_ increased from 1.0 with a high [K^+^] to 2.0 at a low [K^+^]. Snapshots on the right show typical configurations of ions (green) and water molecules (red and white) in the SF (cyan) at the transition states, where *CR*_w–i_ = 2 (upper panel) and 1 (lower panel). The arrows indicate the direction of the outward ion flux from the nanocavity side to the external side

Ion permeation through channels is a passive process simply driven by a transmembrane electrochemical potential gradient, while the permeation mechanism is a highly elaborate process involving strict ion selectivity with a rapid throughput rate [1–4]. In potassium channels, K^+^-selective permeation has been assumed to preferentially bind K^+^ tightly to a selectivity filter (SF) over less permeable Na^+^, excluding Na^+^ binding to the filter from the Na^+^-abundant extracellular solution [5–13]. However, tight K^+^ binding hinders rapid permeation owing to the high energy barriers to be surmounted. Thus, the “knock-on” mechanism was proposed to unite these conflicting tendencies: Upon entering a channel, a K^+^ ion drives out other K^+^ ions that are already tightly bound to the SF [14]. Given the crystal structure of potassium channels, the permeation mechanism is considered more geometrically constrained [15, 16]. The narrow SF does not allow passing of a permeating ion and water molecules with each other (single file), and a short SF with a limited length (12 Å) has four binding sites (*S*_1_–*S*_4_) that can hold up to four to five molecules of either ions or water molecules. Given these constraints, Morais-Cabral et al. proposed a model for K^+^ permeation [15]. Permeating K^+^ ions cannot occupy adjacent binding sites in the SF because of the electrostatic repulsion, and water molecules are intercalated between ions such that K^+^ and water molecules (w) are aligned in alternating arrays, denoted as w–K^+^–w–K^+^ and K^+^–w–K^+^–w. Morais-Cabral et al. considered that the interconversion between the alternating arrays yielded rapid permeation. Molecular dynamics (MD) simulations reproduced the alternating arrays, and the simulation results were interpreted as an incoming ion that forced the expulsion of the downstream ion [17–25] via the three-ion-occupied intermediate (K^+^–w–K^+^–w–K^+^; the “knock-on intermediate” [19]; Fig. 1, right lower snapshot) [15, 16]. This atomistic event is widely considered as a central feature of the knock-on mechanism [17–20, 26].

Despite the appealing simplicity of this mechanism, the following experimental results cast doubt on the knock-on paradigm [27–30]. In a single-file SF, permeating K^+^ ions and water molecules cannot move independently and ion flux is inevitably accompanied by water flux. The number of water molecules carried by one K^+^ ion can be counted across the single-file SF using the streaming potential [31, 32]. The water–ion flux ratio *J*_w_/*J*_i_ [where *J* represents the flux for water molecule (w) and ion (i)] thus evaluated is called the water–ion coupling ratio (*CR*_w–i_) [27, 33]. *CR*_w-i_ values of different types of potassium channels have been measured [27–29, 34, 35], and *CR*_w-i_ values at various K^+^ concentrations (Fig. 1) indicated that the knock-on mechanism is not the exclusive permeation mechanism.

In the knock-on process, the alternating array mode (w–K^+^–w–K^+^ ↔ K^+^–w–K^+^–w) should generate a one-to-one stoichiometry, and a *CR*_w-i_ value of 1 was experimentally confirmed at high K^+^ concentrations ([K^+^] > 0.1 M) (Fig. 1). However, as [K^+^] decreased, *CR*_w–i_ increased gradually from 1 toward 3, which was shared by various potassium channels including hERG, KcsA, and Kir (Fig. 1) [27–29]. The *CR*_w–i_ value also reflects the average number of K^+^ ions within the SF, having a defined number of binding sites. For example, a *CR*_w–i_ of 2, which has been observed at low [K^+^] (< 0.01 M), implies that the SF contains one or two K^+^ ions and the rest of the space is filled with three or two water molecules (e.g., w–w–K^+^–w; Fig. 1, right upper snapshot). This indicates that, at low [K^+^], ions permeate not via the conventional alternating array mode but via sparse ion arrays (sparse array mode; e.g., K^+^–w–w–K^+^ → w–K^+^–w–w → w–w–K^+^-w → …). The *CR*_w-i_ metric provides the ion and water permeation pattern in the SF, and the failure of the canonical (alternating array) mode at low [K^+^] (< 0.01 M) cannot be overlooked since this may threaten the present “knock-on” and “high-affinity” paradigm.

To delineate an alternative permeation mechanism, we sought to clarify the atomistic details of permeation by performing MD simulations of the KcsA potassium channel. For the KcsA channel, there have been numerous studies of the structure and function related to ion permeation [6, 7, 15–17, 20, 26, 28]. In this study, the simulation examined first whether the experimental *CR*_w–i_ values could be reproduced at different [K^+^]. Next, the correlation of ions and water motions within the channel was analyzed using the previously developed event-oriented analysis method [36]. The analysis revealed the rates of ion arrival and release to and from the SF, from which the K^+^ dissociation constant of the channel was obtained. Surprisingly, we found that the K^+^ affinity was unexpectedly low, in contrast to the previously proposed tight K^+^ binding (high-affinity regime), raising questions as to the fundamental assumption about the permeation process [37]. Finally, we propose a queueing mechanism for integrating the alternating and sparse array modes, which provides a general mechanistic framework for potassium channel permeation.

## Results

### MD-simulated ion trajectories across the KcsA potassium channel

**Fig. 2:**
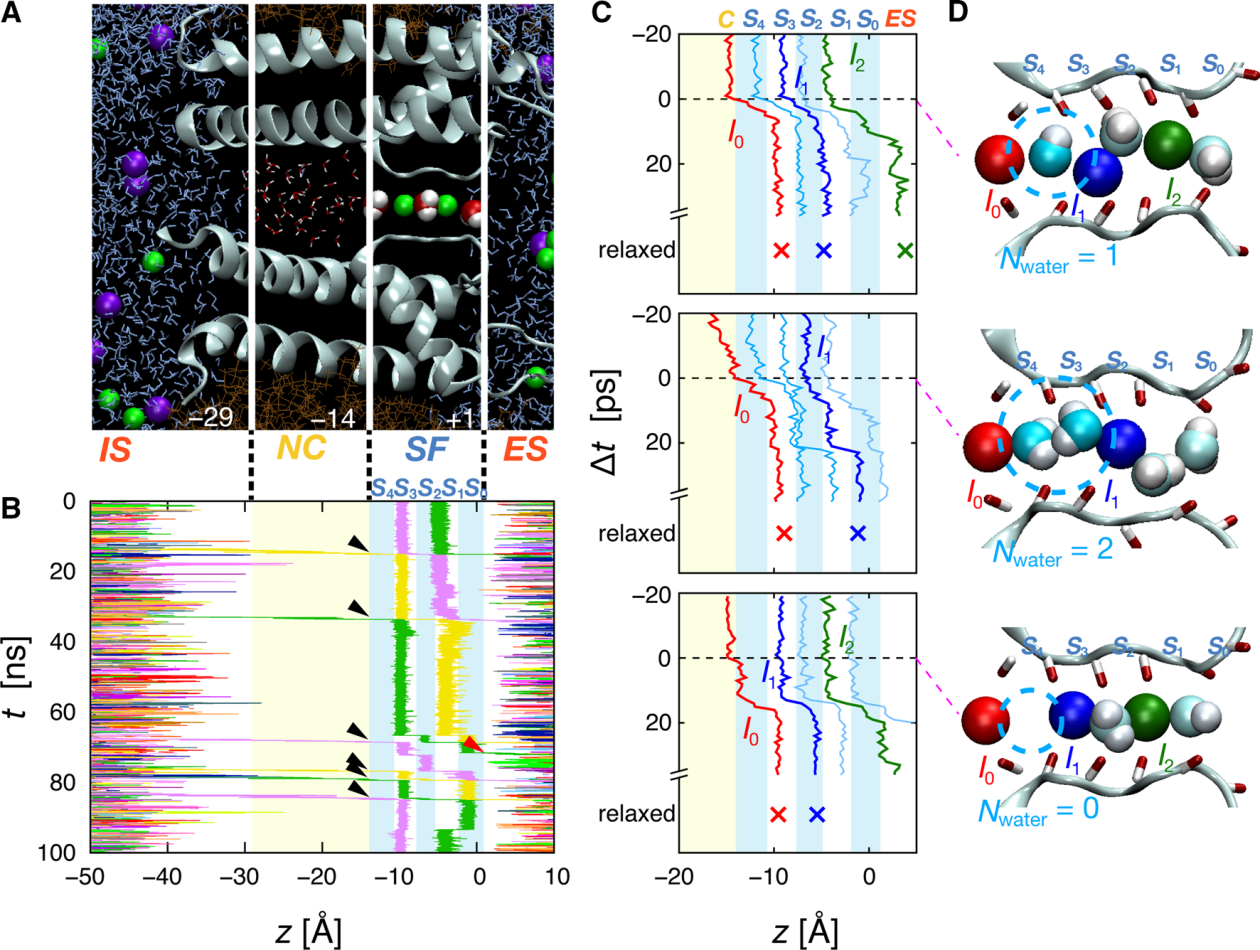
Structure of the KcsA channel and the MD-simulated ion trajectories observed at [K^+^] = 0.15 M. **a** The transmembrane pore domain structure of the KcsA channel (cyan ribbon) in a membrane (brown line). Residues 22–117 in two diagonal subunits are shown to illustrate the inside of the pore. K^+^ and Cl^−^ ions are denoted by green and violet spheres, respectively. Water molecules are shown with a space-filling model (the oxygen atoms are colored in dark red) in the SF, as a stick model in the NC, and as a blue line model in the bulk solution. **b** Trajectories of individual ions (varying colors) permeating through the pore (*z* axis) as a function of time (*t*). The black arrowheads denote SF-in events. The red arrowhead denotes spontaneous release. **c** Ion trajectories aligned to the SF-in event of *I*_0_ (Δ*t* = 0). The positions of the ions after relaxation are indicated by multi symbol. An ion (*I*_0_) undergoing SF-in is shown in red and the preceding ions in blue (*I*_1_) and green (*I*_2_). Water molecules intercalated upon the SF-in of *I*_0_ are shown in sky blue, and the preceding intercalated water molecules are shown light blue. **d** Snapshots of K^+^ and intercalated water molecules (cyan and white) as *I*_0_ undergoes SF-in. Between zero and two water molecules (marked blue circles) were intercalated

The transmembrane domain of the KcsA channel was embedded in a phosphatidylcholine membrane and exposed to various KCl concentrations (Fig. 2a). For most simulations, a positive membrane potential of + 1000 mV was applied (+ 350 mV was also applied and other potential parameters were examined; see Supplementary Table 1), which was high relative to the physiological changes in the membrane potential but relevant to experimental conditions [38–41] and previous simulations for examining permeation properties [18, 19, 36, 42–44]. The outward K^+^ flux was examined because physiologically, potassium channels operate under the application of an outward driving force. The root-mean-square fluctuation of α-carbon atoms in the transmembrane domain was < 2.0 Å [45], indicating that the channel remained in its open conformation throughout the simulation. Moreover, the distances between the α-carbons of the diagonal subunits in the SF were calculated to confirm that the SF was not collapsed (Supplementary Fig. S1). The steady open state allows a continuous ion flux without interruption.

The permeation pathway through the open pore is depicted in Fig. 2a, and a sample MD-simulated ion trajectory along the channel (*z* axis) is shown in Fig. 2b ([K^+^] = 0.15 M). Color traces, denoting individual ions, reveal the progression of ions from the intracellular space (IS) into the nanocavity (NC), followed by progression through the successive sites within the SF before being released into the extracellular space (ES). K^+^ ions frequently access the entrance of the NC, but only a fraction of them enter the NC. Thus, a water-filled NC is mostly empty of ions and transiently holds up to one K^+^ ion (digitalized occupancy) [36]. A hydrated K^+^ ion rapidly traverses the NC and enters the SF by shedding most of the water molecules. In the SF, ions and water molecules progress in a single file from the *S*_4_ to the *S*_0_ site. The number of ions simultaneously occupying the SF was 1–3. During a total simulation time of 2.9 μs, 105 ions were transferred from the IS to the ES. This is equivalent to an electric current of 5.8 ± 1.9 pA, which is roughly comparable with the experimental value observed in the phosphatidylcholine membrane (the single-channel conductance of the KcsA channel in the phosphatidylcholine membrane is approximately one-sixth of that in the phosphatidylglycerol membrane [46, 47]). Concomitantly, 104 water molecules were transferred across the pore, implying *CR*_w–i_ = 0.99 ± 0.10. These data are consistent with experimental measurements in various types of potassium channels exposed to high K^+^ concentrations (i.e., [K^+^] > 0.1 M) such as KcsA, hERG, Ca^2+^-activated K^+^ channel, and Kir2.1 (Fig. 1) [27–29, 35].

To examine the concentration-dependent changes in *CR*_w–i_, we ran MD simulations on three additional K^+^ concentrations (0.05, 0.52, and 1.02 M; Supplementary Table 2). The *CR*_w–i_ values measured from these trajectories are plotted in Fig. 1 (filled red squares), with the previous KcsA experimental results superimposed (red open circles) [28]. These simulations provided the first reproduction of the experimental data, featuring a substantial increase in *CR*_w–i_ at low [K^+^], thus warranting further examination of the microscopic details of the permeation process.

### Ion and water trajectories and distributions at high [K^+^]

We analyzed the permeation process by using the event-oriented analysis method that we developed previously [36]. In the event-oriented analysis, first, an arbitrary event occurring along the MD-simulated ion trajectories was chosen. One such event selected here was the entry of a K^+^ ion into the SF from the NC (Fig. 2a). Figure 2b illustrates six different SF-in events (black arrowheads in Fig. 2b), and three of the time courses were expanded in the picosecond time scale by defining the SF-in moment as the origin of the time axis (Δ*t* = 0) (Fig. 2c) [48]. In Fig. 2c, an SF-in ion is denoted as *I*_0_ (red), and two other ions, which previously underwent SF-in and remained in the SF, are denoted as *I*_1_ (blue) and *I*_2_ (green). The trajectories of the water molecules are drawn in light blue. A snapshot taken at the SF-in moment of *I*_0_ (Δ*t* = 0) (Fig. 2d, upper panel) shows that a water molecule is intercalated between *I*_0_ and *I*_1_ (shown in sky blue). Occasionally, zero or two water molecules are intercalated (Fig. 2d, lower and middle panels) [26].

**Fig. 3:**
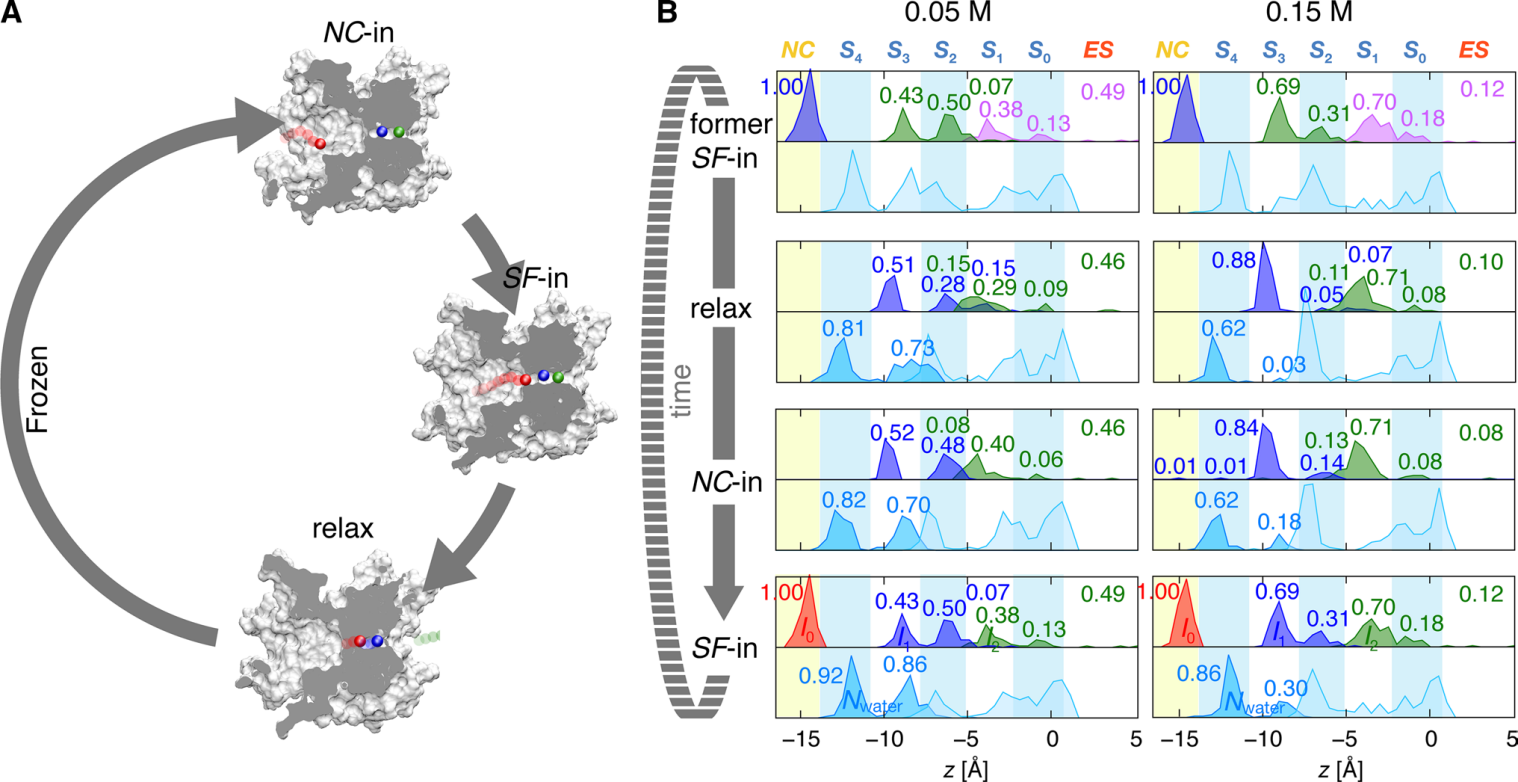
Positions of ions and water molecules during the permeation cycle. **a** A cyclic diagram for K^+^ permeation [36]. Among the permeation events, NC-in and SF-in events followed by relaxation are epochs for characterizing the permeation. After the relaxation, ions and water molecules are immobilized in the SF until the next NC-in (frozen period). In this cyclic diagram, two ions are bared in the SF at the NC-in moment, giving rise to the alternative array mode. Variable single-channel conductances of various potassium channels can be accounted for by the elapsed time of the frozen period governed by the entrance geometry of the NC [74]. **b** Evolution of the ion and water distributions in the SF during the permeation for [K^+^] = 0.05 (left panel) or 0.15 M (right panel). Upper panels: density distributions of *I*_0_ (red), *I*_1_ (blue), *I*_2_ (green), and *I*_3_ (the ion entering ahead of *I*_2_; magenta) along the *z* axis. Lower panels: density distributions of the water molecules, shaded in sky blue for the water molecules between *I*_0_ and *I*_1_ (i.e., upstream from *I*_1_), and in light blue for those downstream from *I*_1_. For the ion distribution, the numbers beside the peaks indicate their integrals, yielding the fractional number of the corresponding ion (*I*_0_ − *I*_3_; upper panels). For example, at 0.15 M [K^+^], 0.69 of *I*_1_ is located at *S*_3_ and 0.31 is located at *S*_2_ at the SF-in moment. For the water distribution, the numbers beside the peaks indicate the absolute number of water molecules intercalated between *I*_0_ and *I*_1_ (lower panels) at a relevant site

The motions of *I*_0_, *I*_1_, and *I*_2_ are strongly coupled [18, 19]. After the SF-in of *I*_0_, the SF transiently holds three ions, forming the three-ion-occupied transition state. This intermediate was alleviated by an exit of the outermost *I*_2_ ion, and ensemble averaging of the SF-in event-oriented trajectories revealed that the intermediate had a brief lifetime of a hundred picoseconds. The remaining *I*_0_ and *I*_1_ then gradually shifted forward to reach sites *S*_3_ and *S*_1_, respectively, eventually forming a “relaxation” state of about 1500 ps after the SF-in (Supplementary Fig. S2). The ion locations in the relaxation state are denoted with a “multiplication symbol” in Fig. 2c. These ion permeation processes repeat periodically and are integrated into a cyclic diagram (Fig. 3a) [36]. Three epoch-making events are depicted with approximate time intervals. Before the SF-in, an ion enters the NC, the moment of which is denoted as NC-in. After the SF-in, the ions and water molecules in the SF reach a stable occupancy state (the relaxation state), after which the ions are immobilized in their position (“frozen”) [36] until the arrival of the next ion at the NC-in moment.

The permeation process is quantitatively evaluated by analyzing the time-lapse distributions of ions and water molecules. Figure 3b shows the distribution of ions (upper panel) and water molecules (lower panel) at three defined moments in the permeation process (event-oriented distribution) at two different [K^+^]. The uppermost and lowermost panels represent the distributions at two successive SF-in moments, and the middle panels show the distribution at the “relaxed” state and the next NC-in moment. The number labeling each peak represents the fractional number of the corresponding ion (*I*_0_ − *I*_3_; Fig. 3b, upper panels) and the absolute number of water molecules intercalated between *I*_0_ and *I*_1_ (lower panels) at the relevant site.

At the SF-in moment at [K^+^] = 0.15 M (Fig. 3B, right top or bottom), *I*_1_ is predominantly located at *S*_3_ (69%) and water molecules upstream from *I*_1_ are mostly located at *S*_4_, where the number of water molecules is 0.86. In single-file permeation, water molecules upstream of *I*_1_ are intercalated between *I*_1_ and *I*_0_ upon the SF-in of *I*_0_. The cumulative sum of water-molecule distributions upstream from *I*_1_ at *S*_4_ and *S*_3_ is counted as *N*_water_ = 0.86 + 0.30 = 1.16 at the SF-in moment. This number of water molecules is driven by the ion flux along the channel, leading to alternating (1:1) ion and water distributions within the *SF*. Thus, the microscopically observed *N*_water_ at the SF-in corresponds to the overall number of water and ion flux ratio during the whole simulation time (*CR*_w–i_ = 0.99) as well as to the experimentally measured *CR*_w–i_ values (Fig. 1). Similar *N*_water_ values were obtained when repeating the simulations for [K^+^] = 0.52 and 1.02 M, being 1.1 and 1.17, respectively (Supplementary Fig. S3). Thus, the alternating array mode predominates above the [K^+^] of 0.15 M.

### Spontaneous release of ions from the SF and the dissociation constant

**Fig. 4:**
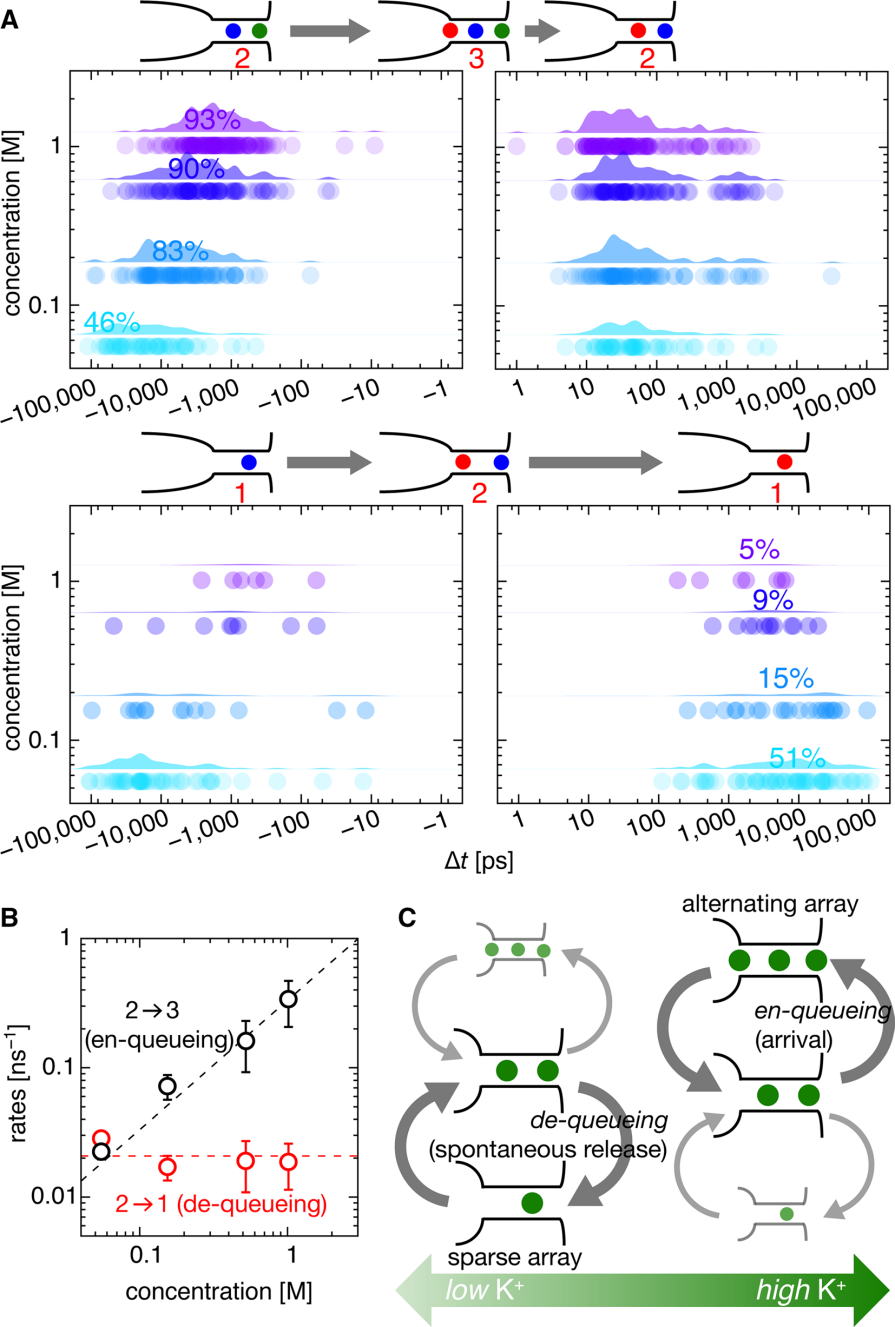
Time spent in spontaneous release, knock-on, waiting periods, and a queueing permeation model. **a** Distribution of entry times to the SF (en-queueing time; left boxes) and exit times of the outermost ion from the SF (de-queueing time; right boxes) for different [K^+^]. Zero time (Δ*t* = 0) is set to coincide with the SF-in for *I*_0_. The dots represent raw data, and the distributions denote their kernel density estimation. The upper panel represents a two-ion-occupied SF before the SF-in, and the lower panel represents a one-ion-occupied SF. **b** Rates from the two-ion-occupied state to the one- (red) and three-ion-occupied (black) states. The black dashed line represents a linear fit for the data (rate = [K^+^]·0.33 ns^−1^), and the red dashed line is a fit calculated from a constant (0.021 ns^−1^). **c** The queueing model. The ions in the SF are shown by green circles. During the long-elapsed time frame for spontaneous release, two water molecules follow and are then intercalated between ions upon the next SF-in, which is measured as *CR*_w–i_ = 2

Noticeable differences in the ion and water distributions were observed at lower [K^+^] (= 0.05 M) (Fig. 3b, left panel). At the SF-in, the number of water molecules located at *S*_3_ was as high as 0.86 and *N*_water_ was approximately 2 (1.78 = 0.92 + 0.86), which is consistent with the experimental *CR*_w–i_ value. More water molecules were intercalated because *I*_1_ shifted outward such that more than half of *I*_1_ was located at *S*_2_ or S_1_ (57% = 50% + 7%). Why was *I*_1_ shifted further downward at low [K^+^]? We found that nearly half of *I*_2_ had already been released out to the ES (49%) before the SF-in, retaining only one K^+^ in the SF. In this case, permeation proceeded without passing through the three-ion-occupied knock-on intermediate, posing a question as to the prerequisite role of the transition intermediate for permeation. After the SF-in, two ions (*I*_0_ and *I*_1_) shifted gradually until the relaxed state, during which half of *I*_1_ was spontaneously released (Fig. 3, left “relax” panel). This spontaneous release was frequently seen in the ion trajectories at 0.05 M (Supplementary Fig. S4). Unexpectedly, spontaneous release was also seen even at 0.15 M (red arrowheads in Figs. 2b, 4a, and Supplementary Video 1), 0.52 M, and 1.02 M (Supplementary Fig. S5). At 0.15 M, 12% of *I*_2_ exited to the ES before the SF-in. Lower ion occupancy in the SF led to greater water molecule occupancy in the SF, which yielded a high *CR*_w–i_ value. The spontaneous release of *I*_2_ before the entry of *I*_0_ marks a significant departure from the conventional knock-on paradigm. Accordingly, we call this permeation as the sparse array mode, which appeared more or less in all the concentration ranges examined.

After the ion and water motions ceased (the frozen state), the distribution remained until the next NC-in moment (Fig. 3; Supplementary Fig. S3 at 0.52 M and 1.02 M) [36]. The period from the relaxed moment to the NC-in moment took up most of the permeation cycle time (92% at 0.15 M), which was expressed as a long frozen period [36]. Thus, the distribution at the relaxed state represents a steady distribution of ions and water in the SF. At 0.05 M, the probability of *I*_2_ occupancy in the SF decreased to ∼ 0.5, indicating that the dissociation constant *K*_d_ was ∼ 0.05 M (see Supplementary Fig. S6). Although this affinity represents an attribute of the outermost ion in the two-ion-occupied state rather than an attribute of a specific site in the SF, this value is consistent with the experimental *K*_d_ values of 0.009–0.050 M [30, 49–52], while a very low value of 0.0004 M was also reported by Lockless et al. [13]. The occupancy of the outermost ion in the SF was equilibrated with the bulk ES with a low affinity, leading to a spontaneous release. In contrast to the assumption in earlier studies that the K^+^ affinity for the SF should be high, the knock-on motion postulating strong ion–ion repulsion is not necessary for permeation.

### De-queueing and en-queueing rates

We show that both the alternating and the sparse array mode existed at all the concentration ranges examined. To examine the transitions between two modes, we consider the permeation as a queueing process, in which permeating ions are in a waiting line to be processed [53, 54]. The exit of the outermost ion into the ES (release) is expressed as de-queueing and the arrival of an incoming ion into the SF (arrival) is en-queueing. When de-queueing, or spontaneous release, occurs earlier than en-queueing, the sparse array mode proceeds. When the sequence of events is reversed, the alternating array mode proceeds. Such considerations of the queueing process provide further quantitative evaluation for the permeation mechanism.

In the alternating array mode, a third ion enters the SF while with two ions in the SF (en-queueing, 2 → 3, where the number shows that of ions in the SF hereinafter; Fig. 4a, left upper panel) and then the outermost ion exits (de-queueing, 3 → 2; Fig. 4a, right upper panel). The transition of 2 → 3 occurs after a long waiting period [36]. Then, 3 → 2 represents the “knock-on” process, constituting a permeation cycle of 2 → 3 → 2. In the sparse array mode, one of two ions in the SF spontaneously exits to the ES, leaving only one ion in the SF (de-queueing, 2 → 1; Fig. 4a, right lower panel). Then, a second ion enters the SF (en-queueing, 1 → 2; Fig. 4a, left lower panel), constituting a 2 → 1 → 2 permeation cycle. Accordingly, it is critically important to know which of the transitions from the two-ion-occupied state happens earlier to either de-queueing or en-queueing.

Now, a quantitative evaluation of the MD-simulated trajectories was performed. First, the exit (de-queueing) time was quantified (Fig. 4a, right panels). Upon the SF-in, the SF holds either two (*I*_1_ and *I*_2_, upper panel) or one (*I*_1_, lower panel) ion, and an event-oriented analysis was performed for these two separate cases at different [K^+^]. From the collection of the trajectories, the elapsed time from the SF-in to the release of either *I*_2_ (upper panel) or *I*_1_ (lower panel) was plotted as dots (Fig. 4a; all the data are shown in Supplementary Fig. S7). The distribution chart above the dots shows the kernel density estimation, deduced from the elapsed time, giving a coarse approximation of the probability density function for the de-queueing time (see “Materials and methods”). *I*_2_ typically exited the channel within 100 ps of *I*_0_ entering the SF (3 → 2: upper right panel), and this de-queueing time is invariable for different concentrations. This immediate alleviation from the transient three-ion-occupied intermediate was expected to be a knock-on scenario, while the time course was first elucidated in this simulation. Another type of de-queueing is spontaneous release (2 → 1: lower right panel), which is significant here for the sparse array mode to proceed. This de-queueing occurs long after the en-queueing (SF-in), with an elapsed time of 16,000 ps at 0.05 M, which is in dramatic contrast with the de-queueing time for the 3 → 2 transition. Hence, such long-overdue time for de-queueing is poorly expressed as the coupling between en-queueing and de-queueing, and the exit process of 2 → 1 is accordingly called the spontaneous release process. During the period of spontaneous release, *I*_0_ advances deeper and more water molecules follow, providing higher *N*_water_ or *CR*_w–i_ values. This mode is frequently seen at the lower concentration of 0.05 M, occurring as high as in half of the cases (51%). Even at high concentrations, the sparse array mode was observed, though infrequently (5% at 1 M).

Next, the arrival (en-queueing) time was considered (left panels). The en-queueing occurred upon the SF-in event, and, thus, the en-queueing time could be read with the elapsed time from the less ion-occupied state to the SF-in, which is shown as dots and as the kernel density estimation (2 → 3: upper panel; 1 → 2: lower panel). The average time for en-queueing of 2 → 3 (upper left panel) gradually prolonged as [K^+^] decreased. The average en-queueing time was 2800 ps for 1 M and increased to 24,200 ps for 0.05 M. This period involves a long waiting (or “frozen” [36]) period as well as the ion passage time across the *NC*; the values correlated well with the average permeation time at different [K^+^] (3700 ns at 1.0 M and 64,500 ns at 0.05 M), which are the inverse of the current amplitudes. The en-queueing times for 1 → 2 seemed to be similar to those for 2 → 3 because the arrival time of the ion at the SF was independent of whether the SF occupied either one or two ions.

### Permeation through the queueing process

Now that the elapsed time for the en-queueing and de-queueing had been evaluated, the dwell time of the two-ion-occupied state can be evaluated. The time from the initiation of the two-ion-occupied state to the three- (en-queueing) or one-ion (de-queueing) occupied state depicts the dwell time of the two-ion-occupied state (*τ*_2_). The value of *τ*_2_ was 11,100 ps at 0.15 M and 19,700 ps at 0.05 M. According to the probability theory, *τ*_2_ is independent of whether the next transition reaches either the three- or the one-ion-occupied state, while examining branching transitions (2 → 3 vs. 2 → 1) provides a relative preference for a destination state. Figure 4a shows that, at 1.02 M, 93% of the transitions reached the three-ion-occupied intermediate (2 → 3; upper panel) and only 5% reached the one-ion-occupied state (2 → 1; lower right panel). In contrast, at 0.05 M, only 46% exhibited the 2 → 3 transition and 51% of the transitions reached the one-ion-occupied state. Three parameters involving *τ*_2_, the separate transition rates (*k*_23_, 2 → 3 en-queueing rate; *k*_21_, 2 → 1 de-queueing rate), and the relative preference are related as follows (Fig. 4c):

1 $$\tau_{2} = \frac{1}{{k_{21} + k_{23} }} = \frac{1}{{k_{21} \left( {1 + N_{23} /N_{21} } \right)}},$$

where *N*_21_ and *N*_23_ are the observed number of transitions for 2 → 1 and for 2 → 3, respectively, and a relation (*N*_23_/*N*_21_ = *k*_23_/*k*_21_) was used. Accordingly, the separate transition rates of *k*_23_ and *k*_21_ were obtained by counting *N*_23_/*N*_21_ (Fig. 4b). For example, at 0.15 M, the *N*_23_/*N*_21_ ratio was 4.2 and *k*_21_ was 0.017 ns^−1^.

Now, the en-queueing rate (*k*_23_, 2 → 3) and the de-queueing rate (*k*_21_, 2 → 1) were compared (Fig. 4b). The de-queueing rate (red) was nearly constant, while the en-queueing rate (black) was proportional to the concentration. Accordingly, as [K^+^] decreased, the en-queueing rate decelerated until the de-queueing rate overwhelmed the en-queueing rate and the sparse array mode predominated. Thus, the two apparent modes of alternating and sparse arrays reflect the relative rates of en-queueing and de-queueing (Fig. 4c).

Lastly, the *K*_d_ value for the two-ion-occupied state was also estimated by these two rates. At the dissociation constant, the en-queueing rate was equal to the de-queueing rate (Fig. 4b). Thus, a ratio, *k*_21_/*k*_23_, gives the *K*_d_ value, which was evaluated to be ∼ 0.06 M. Therefore, the low K^+^ affinity was confirmed.

## Discussion

In this study, using the molecular dynamics (MD) simulation applied to the KcsA potassium channel, we revealed a sparse array permeation mode. With the previously established alternating array mode, the permeation processes were integrated into the queueing mechanism under the low-affinity K^+^ regime. The ion permeation process is dynamic in its nature, and the experimental *CR*_w–i_ values measured under steady-state permeation [27–29, 34, 35], rather than as equilibrium binding data, were used as a benchmark. The transport of water molecules provides crucial insights into the permeation process given the limited length of the SF with a single-file geometry. Our event-oriented analyses of ion and water-molecule trajectories illustrate the underlying atomistic events resulting from the ion-water coupling, which contradicts the conventional knock-on mechanism.

The permeation process is governed by the following principles: (i) the affinity of the outermost ion in the SF is low, and, thus, dynamic equilibrium with respect to the ES governs the occupancy of that ion (Fig. 3b); (ii) the de-queueing rate is constant, but the en-queueing rate linearly depends on [K^+^] (Fig. 4b); (iii) when the spontaneous release (de-queueing) rate exceeds the arrival (en-queueing) rate, the sparse array mode appears, whereas the alternating array mode emerges when the arrival rate is faster than the release rate (Fig. 4b, c); (iv) both the alternating and the sparse mode coexist throughout all the concentration ranges (0.05–1.02 M), and the emergence of either case is stochastic (Fig. 4c). Accordingly, the ion permeation is governed by the queueing process [53, 54].

Under the framework of the general queueing theory [53, 54], permeation through the KcsA channel can be expressed as a queue waiting for service or permeation. The queue length is limited up to three K^+^ ions (with intercalated water molecules) because of the geometrical length limit. At low [K^+^], ion arrival is infrequent and queueing processing in the SF (or de-queueing) is rapid (relative to arrival) enough to maintain a short queue. As the frequency of arrival ions increases, a fraction of arriving ions are not allowed to enter the channel because of the limited queue capacity. When the queue elongates up to three ions, the processing rate of the queue is accelerated.

**Fig. 5:**
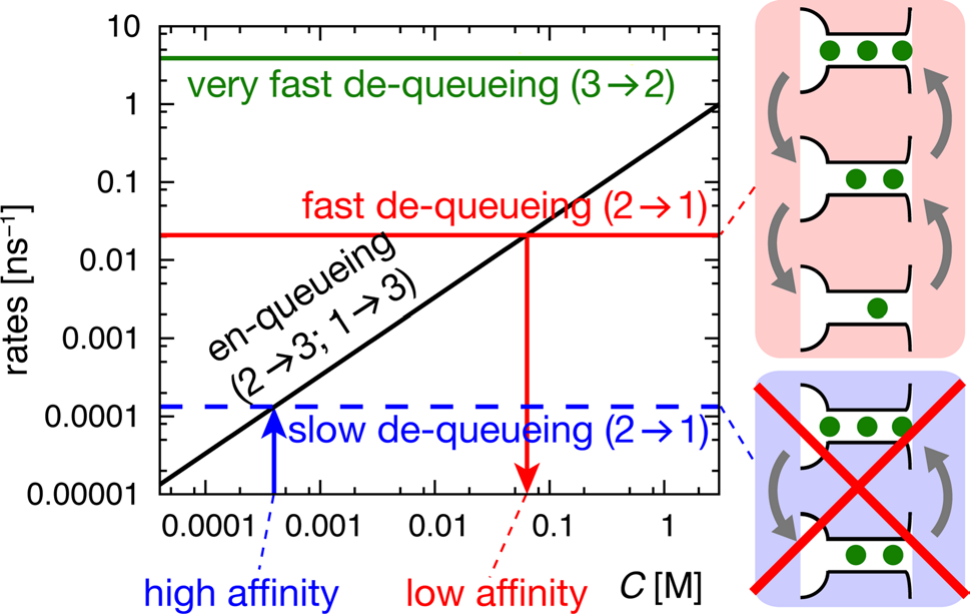
Graphical representation of the queueing mechanism with comparison to the conventional knock-on mechanism. The concentration-dependent en-queueing rate (black line) and the concentration-independent de-queueing rate (2 → 1; red line) are depicted from Fig. 4b. From the intersection of two lines, the K^+^ affinity was deduced (red arrow). The 3 → 2 de-queueing rate is also shown as a green line deduced from Fig. 4a. Given the low K^+^ affinity, the transition from the two-ion-occupied state occurs as either en-queueing or de-queueing in a probabilistic manner. This is the queueing mechanism under the low-affinity regime (right upper cartoon). In contrast, the conventional knock-on mechanism assumes a high-affinity regime. For example, if the *K*_d_ value is 0.0004 M (blue arrow), then the de-queueing rate can be deduced (blue broken line). Then, the 2 → 1 de-queueing process never occurs in the physiological concentration range, and the exclusive alternating mode or knock-on mode is allowed (right lower cartoon)

To characterize the queueing mechanism in comparison to the conventional knock-on mechanism, we expanded Fig. 4 to include wider concentration and the rate ranges (Fig. 5), and depicted the en-queueing and de-queueing rates for the other transitions (3 → 2). The en-queueing rate was linear to the K^+^ concentration, whereas the de-queueing rate was constant. In this study, we found that the de-queueing rate of 2 → 1 was high (red line) and the affinity of K^+^ was deduced as 0.05 M at the intersection of the en-queueing and de-queueing rates. This value is consistent with the experimental values (0.009–0.050 M) [30, 49–52, 55]. At [K^+^] below the *K*_d_ value, de-queueing (2 → 1) occurs earlier than en-queueing, which occurs long afterward (1 → 2), giving rise to the sparse array mode. At [K^+^] above the *K*_d_ value, en-queueing precedes de-queueing. Once en-queuing (2 → 3) occurs, it immediately de-queues (3 → 2), yielding the predominant alternative array mode. Here, the rate of 3 → 2 deduced from Fig. 4a is shown (green line). Even though the en-queueing rate and the de-queueing rate intersect at above 1 M, it does not represent the affinity of the outermost ion because the three-ion-occupied state is transient and does not give an equilibrium constant. Still, the outermost ion in the three-ion-occupied state readily exits. According to the graph, the transition of either 2 → 1 or 2 → 3 bifurcates into the sparse or alternate mode in a concentration-dependent and probabilistic manner (Fig. 5, right upper cartoon).

In contrast, as stated by the knock-on hypothesis, high-affinity K^+^ binding (< 0.001 M) is implicitly assumed to be much below the physiological extracellular [K^+^] (0.003–0.005 M), from which the de-queueing rate is estimated as the intersection with the en-queueing rate (blue arrow; Fig. 5). In this case, the de-queueing rate should be very slow (blue broken line), and spontaneous release cannot be expected. Thus, acceleration by an incoming ion is considered as a prerequisite for surmounting the high energy barrier from the high-affinity site. This leads to an exclusive alternating array mode, yielding a constant *CR*_w–i_ value of 1 over the concentration range, although it is contradictory to the experimental data.

Stoichiometry of ion and water binding to the channel being nearly one to one has been reported, though it has not been performed at lower [K^+^]. In an MD simulation of the KcsA channel, Bernèche and Roux reported that ions and water molecules stably reside in the SF in an alternate array at zero membrane potential [56], indicating a *CR*_w–i_ value of 1. Jensen et al. also showed that *CR*_w–i_ was ∼ 0.9 for Kv1.2 and ∼ 0.5 for Kv1.2/2.1 [19, 44]. On the other hand, Köpfer et al. [26] reported that *CR*_w–i_ was 0 (no water permeation), using the same potential and channel species as the ones we used, although the driving force was imposed by the concentration gradient. A recent paper by Kopec et al. showed that the *CR*_w–i_ value through the MthK channel was also 0 at 220–280 mV, and was ∼ 0.1 at ∼ 900 mV [57]. On the basis of 2D-IR spectral data, however, Kratochvil et al. ruled out adjacent occupancy of ions in the SF [20]. These issues are open for further studies in the future.

The spontaneous release has been overlooked or masked because most simulations have been performed at high K^+^ concentrations [17, 18]. It is noteworthy that spontaneous release was observed in a previous study, although not mentioned in the relevant paper [44]. Ion permeation through the Kv1.2/2.1 channel using the CHARMM potential (we employed the AMBER force field) demonstrated the spontaneous release process when the ion parameter of Bernèche and Roux was employed. The number of ions in the channel (Fig. 2d in Ref. [44]) was below 2 (denoted as “weak K^+^−O” in the paper; voltage < 200 mV), which implicates the presence of the spontaneous release process (de-queueing or 2 → 1 transition). A close inspection of the free energy surface (right panel of Fig. 3c in Ref. [44]) further confirms the presence of the spontaneous release process: there exist a basin at the two-ion center at −16 Å (meaning that there is one ion in the SF) and a leaving ion at 10 Å (meaning that it is located in the ES), indicating that, before an incoming ion reaches the SF, one of the two ions in the SF has already flowed out to the bulk. Thus, the spontaneous release process is likely to be general in potassium channels under simulations irrespective of the choices of parameters.

One may think that spontaneous release is driven by membrane potential. However, that is not considered to be the case. Although a high transmembrane voltage, such as the 1000 mV applied in our simulations, serves as a strong driving force for rapid permeation, the spontaneous release process was observed even at weaker voltages such as 350 mV and at 1.02 M (Supplementary Fig. S5), where spontaneous releases rarely occur (as noted above, in Ref. [44], it was seen at < 200 mV). At 350 mV, the ions sometimes permeate without intervening water molecules (Table S1), and this behavior is consistent with the observation in a recent paper in the case of the MthK channel [57]. Experimentally, the *K*_d_ values have been obtained exclusively at 0 mV (0.009–0.050 M) [30, 49, 50]. On the other hand, our estimated *K*_d_ value (0.05 M) was obtained at 1000 mV, which corresponds to the upper limit or within several times that of the experimental values. This means that the voltage has very little effect on the rate of the 2 → 1 transition. Consequently, the membrane potential is unlikely to be the principal driving force for spontaneous ion release.

The *K*_d_ value assigned to the outermost ion in the *SF* is above the physiological extracellular K^+^ concentration (0.003–0.005 M), and, thus, the occupancy of the relevant ion should be low. Accordingly, under a physiological concentration gradient of [K^+^] across the membrane, more fractions (> 15%) of permeation should occur via the sparse mode.

Our analysis of ion permeation and the proposed queueing permeation mechanism raise a question about the arguments for the mechanisms underlying potassium channel ion selectivity by suggesting that the conventional assumption of a high K^+^ affinity [5–7] is invalid. Future studies should address the interaction processes involving Na^+^ in the SF after binding, which underlies ion selectivity.

## Methods

### System preparation

The system at [K^+^] = 0.15 M was composed of one KcsA channel, 119 dioleoylphosphatidylcholine (DOPC) lipids, 30 K^+^, 22 Cl^−^, and 10,520 water molecules. First, the DOPC bilayer was equilibrated with the surrounding KCl solution for 10 ns. MD simulation was performed using the NPT ensemble (1 bar, 310 K) with a Berendsen’s barostat [58]. The area per lipid after equilibrium was confirmed to be the same as previously reported [59]. The DOPC and the solution overlapping with the pore domain (residues 22–125) of the KcsA channel and the X-ray structure (PDB code: 1K4C) [15] with F125 added to the C-terminal end were removed to incorporate the channel. E71 was protonated as in the previous study [17]. H25, E118, E120, and H124 were also protonated to adapt to the structure in the open state under acidic conditions (pH 4.0). The detailed method for making the open structure has been described previously [45]. The resultant open structure was compared with the atomic force microscopy measurements [60, 61]. An equilibrium simulation was performed for 10 ns using the NPT ensemble (1 bar, 310 K) in which α-carbons were under the influence of a weak harmonic constraint [0.25 kcal/(mol Å^2^)]. Simulation of the equilibration using the NVT ensemble (310 K) with a Berendsen’s thermostat [58] continued for 10 ns after the constraint on the KcsA channel was removed. To make the initial configuration at different concentrations, we randomly chose the water molecules and replaced them with K^+^ and Cl^−^; the system was then equilibrated.

### Molecular dynamics simulations

The following empirical potentials were used: the TIP3P model for water, ff94 force field for the channel, Dang model for the K^+^ ions, and Siu model for the lipids [59, 62–64]. The SPC/E model for water and the ff99SB force field for the KcsA channel were also examined (see Table S1) [65, 66]. Ten or 50 independent initial coordinates were generated to minimize the dependency of the initial configurations and evolved over time according to Newton’s equations of motion. Several types of ion and water array in the SF (K^+^–w–K^+^–w, w-K^+^–w–K^+^, K^+^–w–w–K^+^, and K^+^–V–K^+^–w, where V stands for vacancy) were generated as initial configurations. The initial equilibrium runs, including the ion and water molecule permeation events in the process, were omitted from the analyses.

MD simulations were performed at a constant volume (78.0 × 81.2 × 85.0 Å^3^) and a constant temperature (310 K) using the Berendsen’s thermostat [58]. Periodic boundary conditions were imposed. Long-range interactions were calculated using the particle–mesh Ewald method [67] with an 8-Å real-space cut-off. The bonds, including those for the H atoms, were constrained using the SHAKE algorithm [68] to enable a time step of 2 fs.

The SANDER module of the AMBER11 package was modified to apply an electric field and was used to observe passive ion transport [69]. The electric field was applied to mimic a voltage-clamp experiment [70]. A voltage of 800 mV was applied to the SF (14 Å) and 200 mV was applied to the NC + IS (19 Å = 15 Å in the NC + 4 Å in the IS) (Fig. 2a) [71]. MD simulations were performed over 130 ns for 10 configurations and over 40 ns for 40 configurations, where [K^+^] = 0.15 M.

The SF-in moments were defined as the moment when an ion in the NC entered the SF and reached S_4_ [36]. Similarly, the NC-in moments were defined as the moment when an ion in the IS entered the NC. Additionally, the probability of finding an ion at each site was calculated using the same method that was applied in Ref. [36].

### Kernel density estimation

To estimate the probability density of the de-queueing and en-queueing time, we used the kernel density estimation [72]. As the kernel function, a Gaussian function was used:

$$f\left( {\Delta t} \right) = \frac{1}{nh}\sum\limits_{i = 2}^{n} {\frac{1}{{\sqrt {nh} }}e^{{ - (\Delta t - \Delta t_{i} )^{2} /h}} } ,$$

where *f*, *h*, *n*, and Δ*t*_*i*_ are an unknown density, a smoothing parameter called bandwidth, the quantity of the data, and each data point (shown by dots in Fig. 4a), respectively. For the value of *h*, 1/*n* was employed.

### Snapshots and videos

All snapshots and videos were produced using the Visual Molecular Dynamics package [73].

### Electronic supplementary material

Below is the link to the electronic supplementary material.

Supplementary material 1 (DOCX 996 kb)

Supplementary material 2 (MP4 7421 kb)
