# Supplementary material for: Queueing arrival and release mechanism for K+ permeation through a potassium channel
Source: J Physiol Sci. 2019 Aug 27;69(6):919–30. doi: 10.1007/s12576-019-00706-4 (PMC10717923; doi:10.1007/s12576-019-00706-4)
Supplement: Supplementary file 1 — Supplementary material 1 (DOCX 996 kb) [file 12576_2019_706_MOESM1_ESM.docx]

Supporting Information

K^+^ permeation through a potassium channel governed by queueing arrival and release

Takashi Sumikama, and Shigetoshi Oiki*


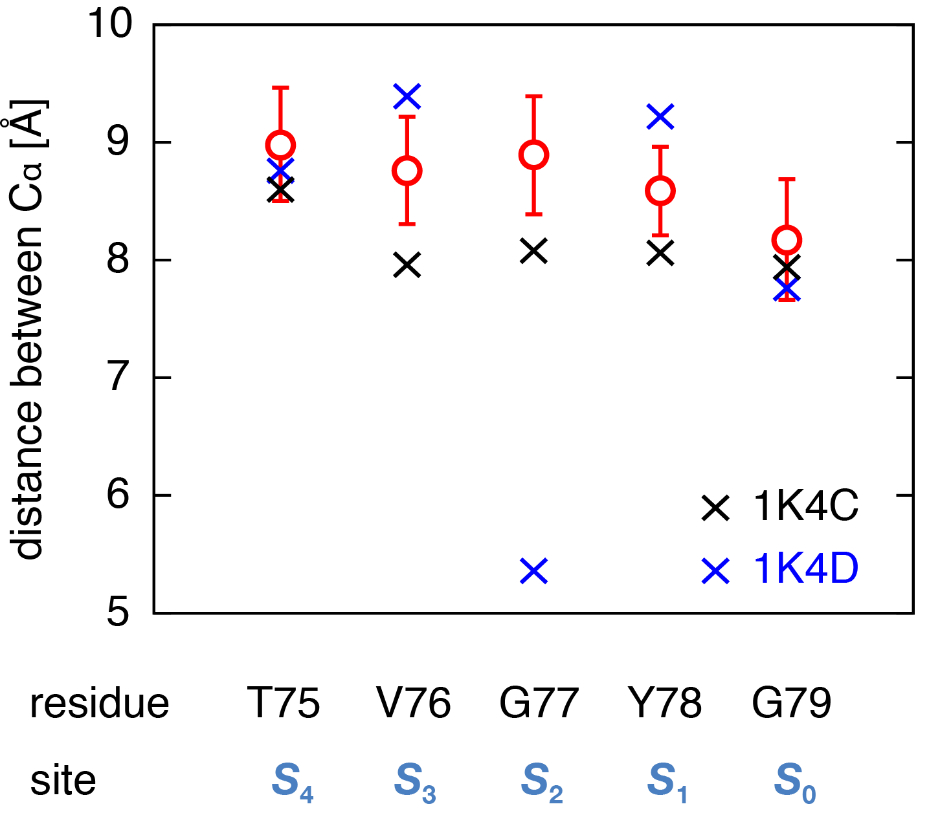


**Figure S1.** Average distance between the α-carbon atoms of the inter-subunits. The standard deviation of each data is shown by the error bars. The distance between the α-carbon atoms of the inter-subunits of the X-ray crystal structure (1K4C and 1K4D) is indicated by an “×”.

**
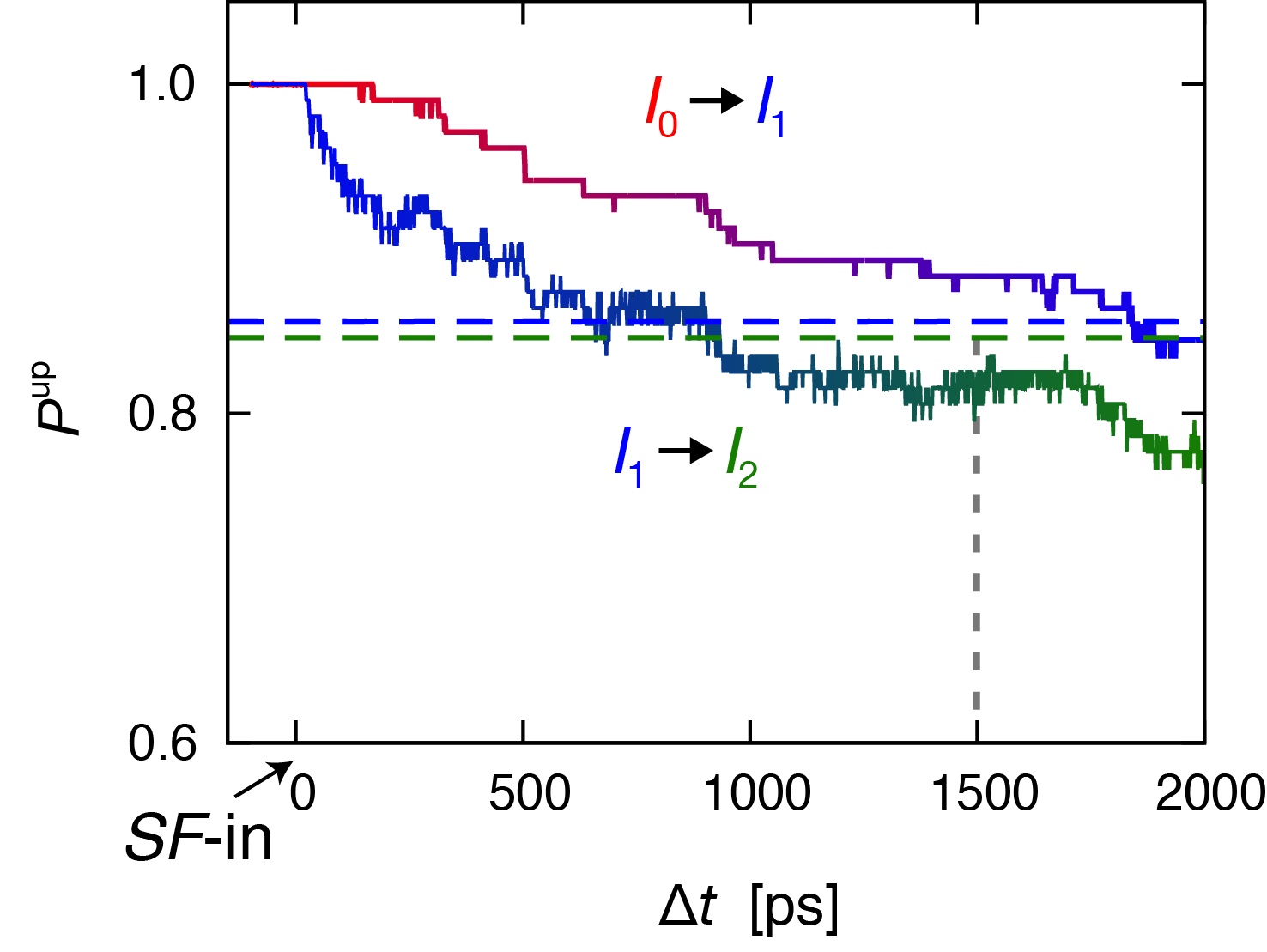
**

**Figure S2.** Illustration of the relaxation process of ions in the SF from the SF-in moment at 0.15 M. The location of the pore is divided into upstream and downstream positions, and for *I*_0_ the upstream positions are the NC, S_4_, and S_3_ sites, and for *I*_1_ the upstream positions are the S_4_, S_3_, S_2_, and S_1_ sites. The probability of an ion being located in the upstream position is defined as *P*^up^. Upon the SF-in, *I*_0_ is located at S_4_; thus, *P*^up^ is 100% (red trace). *I*_0_ moves downstream and leaves the upstream position with gradually decreasing *P*^up^. The same is true for *I*_1_, except that it moves downstream more quickly (blue trace). Meanwhile, an ion enters the SF and the previous *I*_0_ turns into *I*_1_, which is represented by the gradual change in color in the trace (red 🡪 blue). Similarly, *I*_1_ turns into *I*_2_ (blue 🡪 green). The blue and green dashed horizontal lines show the probability of finding *I*_1_ at the NC, S_4_, and S_3_ sites at the NC-in moment and the probability of finding *I*_2_ at the S_3_, S_2_, and S_1_ sites at the NC-in moment, respectively. The *P*^up^ of *I*_0_ to *I*_1_ and the *P*^up^ of *I*_1_ to *I*_2_ are very close to the probability of finding *I*_1_ at the NC, S_4_, and S_3_ sites at the NC-in moment and to the probability of finding *I*_2_ at the S_3_, S_2_, and S_1_ sites at the NC-in moment, respectively, at 1,500 ps after the SF-in. This indicates that *I*_0_ and *I*_1_ respectively replace the positions of *I*_1_ and *I*_2_ in the former permeation event and that the relaxation time is 1,500 ps. The relaxation times at different concentrations were estimated using the same method.

**
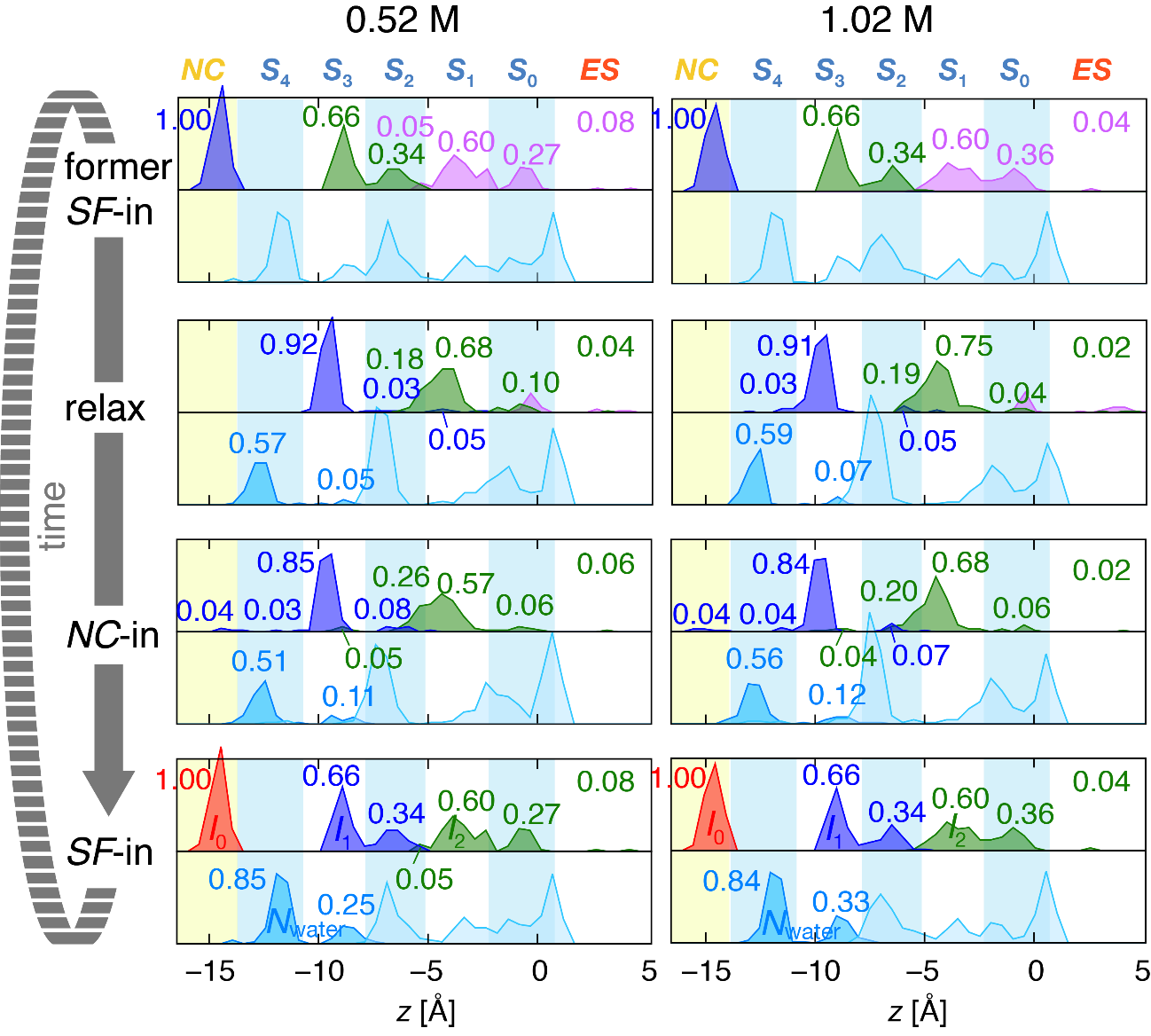
**

**Figure S3.** Ion and water distributions in the SF at different stages in the permeation cycle, determined for [K^+^] = 0.52 and 1.02 M. The figure is identical to Fig. 3 in the main text, except that it shows different concentrations. The distributions of *I*_0_ (red), *I*_1_ (blue), *I*_2_ (green), and *I*_3_ (the ion entering ahead of *I*_2_; magenta) are shown along the *z*-axis (upper panel); the water-molecule distributions are shown underneath. The distribution of water molecules between *I*_0_ and *I*_1_ (upstream of *I*_1_) is shown in sky blue and that of other water molecules downstream of *I*_1_ is shown in light blue. The vertical axis shows the density of the ions and water molecules, in which the vertical length of the box is 1 Å^−1^. The numbers beside each peak denote their integrals, indicating the average number of ions and water molecules found at each site.


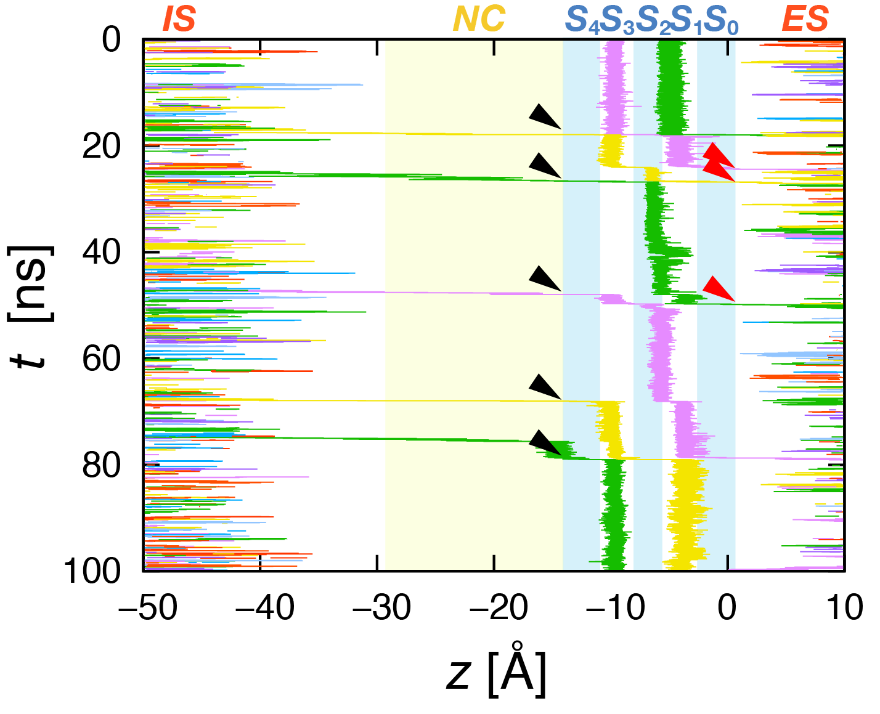


**Figure S4.** Ion trajectories through the KcsA channel at 0.05 M. This is identical to Fig. 2B in the main text, albeit for the concentration. The SF-in moments are shown by the black arrowheads. The red arrowheads denote the spontaneous release of ions from the channel.


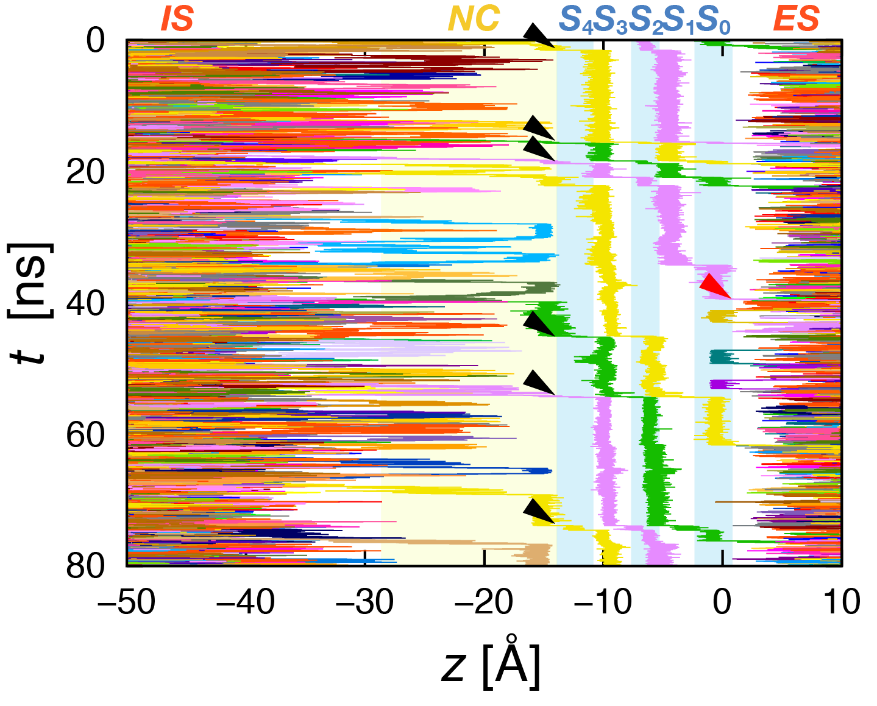


**Figure S5.** Ion trajectories through the KcsA channel at 350 mV and at 1 M (#4 in Supplementary Table 1). This is identical to Fig. 2B in the main text, albeit for the concentration. The SF-in moments are shown by the black arrowheads. The red arrowheads denote the spontaneous release of ions from the channel.


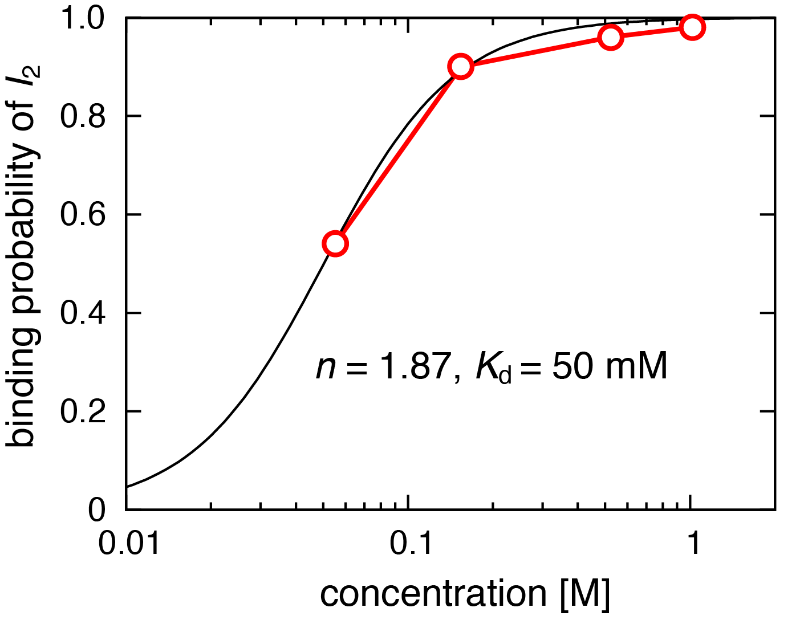


**Figure S6.** Estimation of the dissociation constant *K*_d_. The concentration dependence of the probability of finding *I*_2_ at the S_2_, S_1_, and S_0_ sites (hence, in the SF) at the relaxed state is plotted (red). The fit by the Hill equation (black curve) yields a Hill coefficient of *n* = 1.87 and *K*_d_ = 50 mM.


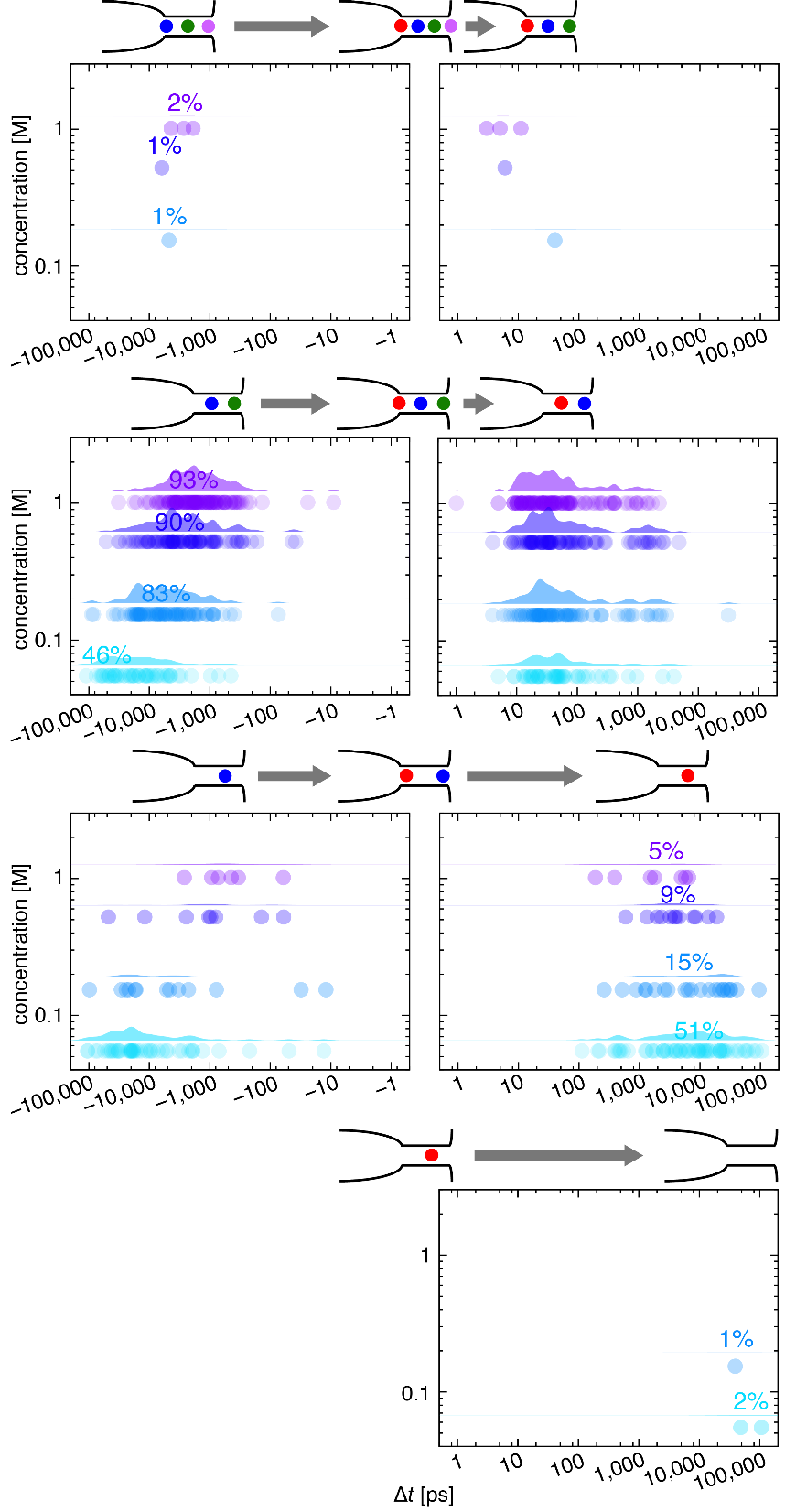


**Figure S7.** Full depiction of the data shown in Fig. 4A in the main text. In this figure, the following three transition times are added: transition from the three-ion-occupied state to the four-ion-occupied state (top left), transition from the four-ion-occupied state to the three-ion-occupied state (top right), and transition from the one-ion-occupied state to the empty state (bottom right).

Table 1. MD simulations conducted at [K^+^] = 1.02 M using different potential parameters for water and the KcsA channel. Bold-font characters denote differences from the main run (#0).

|  | #0 | #1 | #2 | #3 | #4 |
| --- | --- | --- | --- | --- | --- |
| Force fields for water | TIP3P | TIP3P | **SPC/E** | **SPC/E** | TIP3P |
| Force fields for KcsA | ff94 | **ff99SB** | ff94 | **ff99SB** | ff94 |
| Voltages | 1,000 mV | 1,000 mV | 1,000 mV | 1,000 mV | **350** mV |
| Simulation time [μs] | 2.9 | 0.1 | 0.1 | 0.1 | 3.0 |
| Independent simulations | 50 | 10 | 10 | 10 | 50 |
| No. of ion permeations | 105 | 21 | 30 | 21 | 99 |
| No. of water-molecule permeations | 104 | 23 | 33 | 21 | 64 |

**Table 2.** Ion permeation executed at [K^+^] = 0.05, 0.52, and 1.02 M. The applied force fields and voltage were the same as those of the main run executed at [K^+^] = 0.15 M (#0 in Supplementary Table 1).

|  | **0.05 M** | **0.52 M** | **1.02 M** |
| --- | --- | --- | --- |
| Simulation time [μs] | 6.5 | 0.81 | 0.5 |
| Independent simulations | 50 | 10 | 10 |
| No. of ion permeation | 101 | 109 | 128 |
| No. of water permeation | 141 | 97 | 116 |


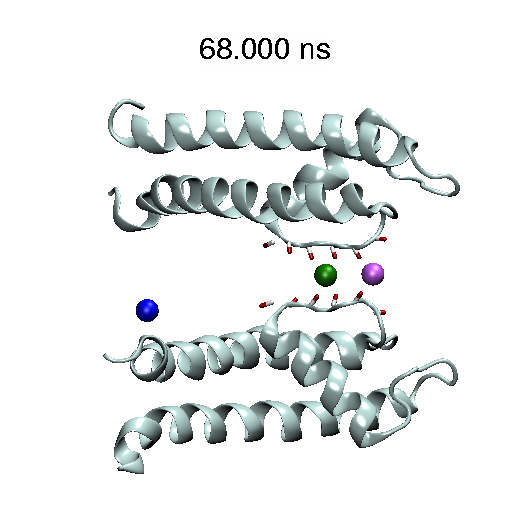


**Video 1.** Video of the spontaneous release motion in Fig. 2B in the main text. The video is from 68 ns to 77 ns. Only the channel and permeating ions are depicted.
